# Effect of genetic liability to migraine and its subtypes on breast cancer: a mendelian randomization study

**DOI:** 10.1186/s12885-023-11337-9

**Published:** 2023-09-20

**Authors:** Tian Fang, Zhihao Zhang, Huijie Zhou, Wanchun Wu, Fuqing Ji, Liqun Zou

**Affiliations:** 1 Department of Medical Oncology, Cancer Center, West China Hospital, Sichuan University, No. 37 Guo Xue Alley, Chengdu, Sichuan 610041 China; 2 Department of Breast Center, West China Hospital, Sichuan University, No. 37 Guo Xue Alley, Chengdu, Sichuan 610041 China; 3 Department of Thyroid Breast Surgery, Xi’an NO.3 Hospital, the Affiliated Hospital of Northwest University, Xi’an, Shaanxi 710018 P.R. China

**Keywords:** Breast cancer, Migraine with aura, Migraine without aura, Any migraine, Mendelian randomization.

## Abstract

**Background:**

The relationship between migraine and breast cancer risk has generated conflicting findings. We attempted to assess the association between migraine and breast cancer risk using Mendelian randomization (MR) analysis.

**Methods:**

We selected genetic instruments associated with migraine from a recently published genome-wide association studies (GWAS). Inverse variant weighted (IVW) analysis was adopted as the main method, and we also performed the weighted-median method and the MR‒Egger, MR pleiotropy residual sum and outlier (MR-PRESSO), and MR Robust Adjusted Profile Score (MR-RAPS) methods as supplements.

**Results:**

Our MR suggested that any migraine (AM) was a risk factor for overall breast cancer (IVW: odds ratio (OR) = 1.072, 95% confidence intervals (CI) = 1.035–1.110, *P* = 8.78 × 10^− 5^, false discovery rate *(FDR)* = 7.36 × 10^− 4^) and estrogen receptor-positive (ER+) breast cancer (IVW: OR = 1.066, 95% CI = 1.023–1.111, *P* = 0.0024; *FDR* = 0.0108) but not estrogen receptor-negative (ER-) breast cancer. In its subtype analysis, women with a history of migraine without aura (MO) had an increased risk of ER- breast cancer (IVW: OR = 1.089, 95% CI = 1.019–1.163, *P* = 0.0118, *FDR* = 0.0354), and MO was suggestively associated with the risk of overall breast cancer (*FDR* > 0.05 and IVW *P* < 0.05). No significant heterogeneity or horizontal pleiotropy was found in the sensitivity analysis.

**Conclusion:**

This study suggested that women with AM have an increased risk of overall breast cancer and ER + breast cancer. MO was suggestively associated with the risk of overall breast cancer and ER- breast cancer.

**Supplementary Information:**

The online version contains supplementary material available at 10.1186/s12885-023-11337-9.

## Introduction

Migraine is a clinically common neurological disorder that is most common in women aged 25–55 [1, 2]. Nowadays, migraine is highly prevalent [3]. Notably, migraine is associated with many poor health outcomes, such as cardiovascular disease [4], dementia [5] and cancer [6]. Therefore, migraine appears to be a major public health problem. Breast cancer is the most prevalent female malignant tumour and poses a huge public health and economic burden [7]. Evidence on the association between migraine and risk of breast cancer is limited and inconsistent [8–12]. Evidence from the Nurses’ Health Studie didn’t find the association between migraine and breast cancer [11]. However, an association between migraine and breast cancer was revealed in another two study [8, 12]. Inconsistent findings and limitations of observational studies, such as undetectable confounders, hamper causal assessment of the relationship between migraine and breast cancer.

Traditional observational studies have inherent shortcomings in exploring casual links between exposure and outcome. Mendelian randomization (MR) studies, a new approach to epidemiological research, allow the use of large samples of pooled genome-wide association studies (GWAS) data to explore the relationship between risk factors and outcomes [13–15]. MR studies effectively reduce the impact of reverse causality and causal confounding estimates in observed data. In addition, measurement error has less impact on the results due to the high precision of single nucleotide polymorphisms (SNPs) measurement [16]. In addition, many published GWAS provide a rich resource of data [17]. Here, we aimed to estimate the causal relationship between genetic liability for migraine and breast cancer by applying a two-sample MR analysis [18].

## Materials and methods

### Study design

**Fig. 1:**
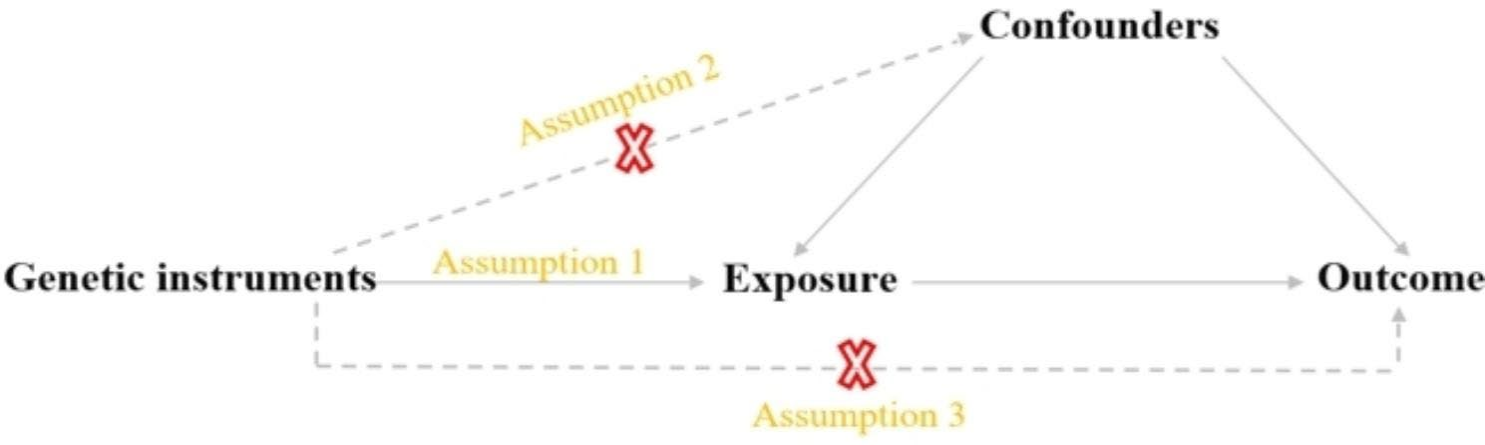
**A**. Basic assumptions of Mendelian randomization. **B**. Flow chart of the analytical methods and how the MR analysis was performed step by step

We conducted a two-sample MR study to examine the causal relationship between exposure and outcome. Genetic variation as an instrumental variable must follow three assumptions: [1] Genetic variations are strongly related to exposure [2]. Genetic variations are not associated with potential confounding [3]. Genetic variations do not influence outcomes directly [19] (Fig. 1).

### Data sources

This MR analysis utilized the largest published GWAS data; all of the participants in those cohorts being of European descent (Table 1). Ethical approval and consent information for the summarized statistics were taken from the original publication.

**Table 1:** Characteristics of genome-wide association studies used in the analyses

Traits	Case/Control	Cohort	PMID
**Migraine**			
Any migraine (AM)	102,084/ 771,257	IHGC2016 (European descent) UKBB (European, British) deCODE (European, Icelandic) DBDS (European, Danish) LUMINA (European, Dutch)	35,115,687
Migraine with aura (MA)	14,624/682,301		
Migraine without aura (MO)	15,055/703,852		
**Breast Cancer**			
Overall BC	122,977/105,974	Oncoarray (European descent) iCOGS (European descent	29,059,683
ER + BC	69,501/105,974		
ER- BC	21,468/105,974		

BC: breast cancer; ER + BC: estrogen positive breast cancer; ER- BC: estrogen negative breast cancer

The data on any migraine (AM) were derived from a recently published article composed of 102,804 cases and 771,257 controls, which included 14,624 cases of migraine with aura (MA) and 15,055 cases of migraine without aura (MO) from five study collections (IHGC2016, UKBB, 23andMe, GeneRISK, and HUNT) after removing overlapping participants. Patients without subtype information were not included in the subtype analysis [18]. The meta-analysis of the study collections was conducted in a fixed-effect model using GWAMA [20]. All study collections were adjusted for sex and more than four leading principal components, and age was conducted as a covariate if it was available in the genetic population [18].

The GWAS summary statistics of breast cancer were derived from the Breast Cancer Association Consortium (BCAC) with 122,977 cases (69,501 estrogen receptor positive (ER+) and 21,468 estrogen receptor negative (ER−)) and 105,974 controls of European ancestry from OncoArray and ICOGS arrays [21]. Overlapping participants were removed from the iCOGS dataset because of the better genomic coverage provided by the OncoArray array. The iCOGS and OncoArray cohorts were adjusted for study and country, respectively [21].

### Selection of instrumental variables

A series of rigorous steps were applied to the screening of single nucleotide polymorphisms (SNPs). First, we selected the SNPs as instrument variants (IVs) of migraine in the threshold of *P* < 5 × 10 − 8, and the *P* was derived from original exposure-GWAS with fixed-model [18]. Second, SNPs were removed if the minor allele frequency was less than 0.01, and linkage disequilibrium (LD) estimates were performed (R2 < 0.001, window size 10,000 kb). Third, the SNPs were removed if they were directly related to the outcome *(P* < 5 × 10 − 8) and the *P* was derived from original outcome-GWAS with fixed-model. Fourth, all candidate SNPs were checked to avoid any possible confounders *(P* < 5 × 10 − 8) by PhenoScanner (http://www.phenoscanner.medschl.cam.ac.uk/phenoscanner); the removed SNPs as possible confounders for breast cancer, such as body mass index (BMI), NSAIDs, age of menarche or age of menopause. The F statistics were used to detect weak instrumental variables, and there were no weak instrumental variables when F > 10.

### Primary MR analysis

The Wald ratio was used to assess the effect of migraine on breast cancer for each SNP. All SNP effects were meta-analysed by the inverse-variance weighted (IVW) method [22]. We performed Cochran’s Q test to check heterogeneity, and the random-effects model of the IVW method was adopted if the heterogeneity existed; otherwise, the fixed-effects model of the IVW method was used as the primary result in our study [23].

### Sensitivity analysis

In sensitivity analyses, MR‒Egger and weighted median (WM) methods were applied to account for horizontal pleiotropic effects. The MR‒Egger method was based on the Instrument Strength Independent of Direct Effect assumption, which often provides imprecise and low statistical power MR results, especially when meeting small sizes of SNPs (e.g., < 10) [24]. In our MR study, MR‒Egger was mainly used to detect pleiotropy; a statistically significant intercept indicates directional pleiotropy [24]. The WM method was more reliable if more than 50% of SNPs were invalid instruments (e.g., due to pleiotropy) [25]. Considering measurement error in the effects of SNP, a newly developed analysis, called the Robust Adjusted Profile Score (MR-RAPS), was performed to reduce bias from weak IV. However, this method was applicable when the sample size of SNPs was greater than 7 [26]. In addition, MR-PRESSO analysis was used to detect outliers, which can reduce heterogeneity by removing those outliers that may lead to heterogeneity [27]. We performed leave-one-out method analysis to determine potentially influential SNPs by removing each SNP. We adjusted the multiple testing by false discovery rate (*FDR*). Summary of the different methods used for MR analysis was shown in Supplementary Table 1.

### MR procedures

We performed three steps for our study to eliminate biased results due to heterogeneity (Supplementary Fig. 1). Step 1: We first conducted MR analysis with all the above-selected SNPs, and then the MRPRESSO outlier test was performed; we went to the second step if the MRPRESSO outliers were excited (*P* < 0.05). Step 2: We reassessed the MR analysis after removing all outliers (*P* < 0.05). Step 3: If heterogeneity was still present, we excluded all SNPs with a *P* value less than 1 in the MR-PRESSO test and reevaluated the MR analysis. Finally, if there were potentially influential SNPs examined by the leave-one-out test, we will explain the results with caution.

The “TwoSampleMR”, “mr.raps” and “MRPRESSO” packages were applied in our MR study. All statistical analyses were performed based on R software 4.1.1.

## Results

Detailed information on the selected SNPs is shown in Supplementary Table 2. The F statistics for all SNPs ranged from 29.9 to 314.8. For AM and MO, most associations were well powered (Supplementary Table 3), For MA, statistical power was lower, we deemed that it might result from low explained variance, as it had only three IVs, suggesting that the relationships between MA and breast cancer should be cautious.

**Fig. 2:**
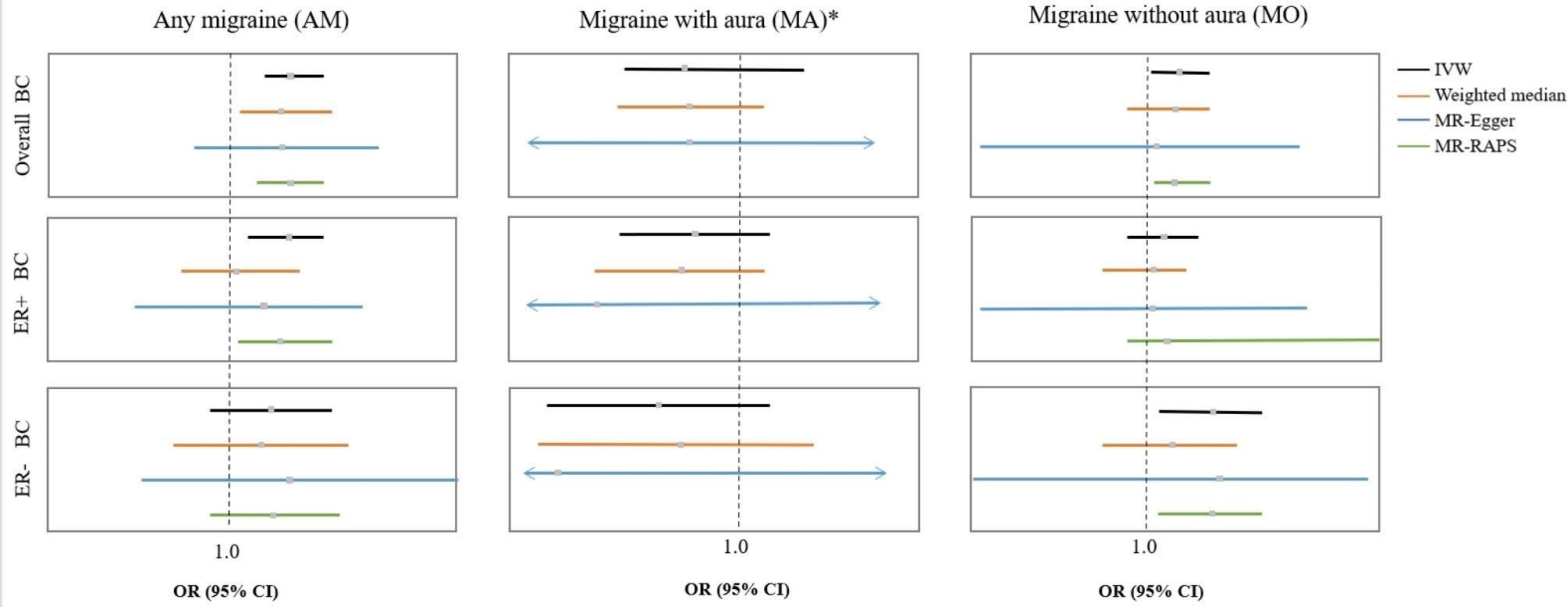
The forest figure for MR analysis. Migraine with aura (MA)*: The MR-RAPS method did not apply to MA (3 SNPs) because this method was applicable when the sample size of SNPs was greater than 7. ER + BC: breast cancer; ER + BC: estrogen-positive breast cancer; ER- BC: estrogen-negative breast cancer; MA: migraine with aura. IVW: inverse variance weighting. MR-RAPS: Robust adjusted profile score. The black line represents the results of the IVW method. The orange line represents the results of the weighted median method. The blue line represents the results of the MR‒Egger method. The green line represents the results of the MR-RAPS method

Four SNPs associated with the confounders for the AM were removed; three of them are related to BMI (rs1472662, rs42854, and rs12708529), and another one (rs1019990) is related to age at menarche. rs10828247 was excluded when the exposure was AM because of the directivity relationship with the outcome *(P* < 5 × 10 − 8). The MR estimates at different steps of the causal effect of migraine on breast cancer from the series of analysis methods are presented in Supplementary Tables 4–6. The last step of MR analysis is shown in Table 2; Fig. 2.

**Table 2:** MR estimates of assessing the causal effect of migraine on breast cancer in IVW method

Outcome	Step^#^	Overall BC IVW method			ER + BC IVW method			ER- BC IVW method		
		NSNP	OR (95%CI)	*P*	NSNP	OR (95%CI)	*P*	NSNP	OR (95%CI)	*P*
AM	3	79	1.072 (1.035, 1.110)	**8.78 × 10** ^**− 5**^	81	1.066 (1.023, 1.111)	**0.0024**	80	1.045 (0.979, 1.115)	0.1871
MA	1	3	0.922 (0.840, 1.103)	0.0919	3	0.939 (0.840, 1.051)	0.2739	3	0.883 (0.745, 1.047)	0.1518
MO	1	12	1.042 (1.005, 1.081)	**0.0267**	12	1.022 (0.979, 1.068)	0.3217	12	1.089 (1.019, 1.163)	**0.0118**

Step^#^: 1, MR analysis with the all remained SNPs; 2, MR analysis after eliminating MRPRESSO outlier (with P < 0.05); 3, MR analysis after removing all the SNPs (with P < 1.00 in MR-PRESSO test); NSNP, number of single nucleotide polymorphism; MR, Mendelian randomization; IVW: inverse variance weighting; OR, odds ratio; CI, confidence interval; BC: breast cancer; ER + BC: estrogen positive breast cancer; ER- BC: estrogen negative breast cancer; AM: any migraine; MA: migraine with aura; MO: migraine without aura; Bold font: The p-values < 0.05 are statistically significant. The ORs were scaled to a 1-unit increase in log-transformed OR of migraine. P values are for ORs (95% CIs)

### The effect of AM on breast cancer

The MR analysis showed that genetically predicted AM (per log-odds ratio increase) was associated with a 7.2% higher risk of overall breast cancer (IVW: OR = 1.072, 95% CI = 1.035–1.110, *P* = 8.78 × 10^− 5^; WM: OR = 1.063, 95% CI = 1.006–1.123, *P* = 0.0283; MR-Egger: OR = 1.062, 95% CI = 0.956–1.179, *P* = 0.2639; MR-RAPS: OR = 1.069, 95% CI = 1.026–1.113, *P* = 0.0015) and a 6.6% higher risk of ER + breast cancer (IVW: OR = 1.066, 95% CI = 1.023–1.111, *P* = 0.0024; WM: OR = 1.010, 95% CI = 0.947–1.078, *P* = 0.7533; MR-Egger: OR = 1.038, 95% CI = 0.902–1.160, *P* = 0.7277; MR-RAPS: OR = 1.064, 95% CI = 1.013–1.117, *P* = 0.0128). However, no causal effect of AM on ER- breast cancer was found (IVW: OR = 1.045, 95% CI = 0.979–1.115, *P* = 0.1871; WM: OR = 1.037, 95% CI = 0.940–1.144, *P* = 0.4728; MR-Egger: OR = 1.077, 95% CI = 0.880–1.319, *P* = 0.4714; MR-RAPS: OR = 1.059, 95% CI = 0.982–1.142, *P* = 0.1357). The effect estimations from various sensitive analysis all pointed to the same direction, which indicated that the results are robust. An additional, adjusted *P* value of IVW after *FDR* was corrected suggested that AM was still a risk factor for overall breast cancer (*FDR* = 7.36 × 10^− 4^) and ER + breast cancer (*FDR* = 0.0108). Detailed information on the MR analysis for different steps is shown in Supplementary Table 4.

No horizontal pleiotropy was detected in this part of the MR analysis (Table 3). Heterogeneity was eliminated after performing Step 3 MR estimate (MR analysis after removing all the SNPs whose *P* value was less than 1 after the MRPRESSO test). Moreover, the leave-one-out test showed that there were no potentially influential SNPs in this part (Supplementary Fig. 2). Therefore, we can draw a robust conclusion.

**Table 3:** Heterogeneity and horizontal pleiotropy analyses results

Outcome	AM		MA				MO	
	P_(Heterogeneity)_	P_(Pleiotropy)_	P_(Heterogeneity)_		P_(Pleiotropy)_		P_(Heterogeneity)_	P_(Pleiotropy)_
Overall BC	0.084	0.846	0.092		0.998		0.347	0.300
ER + BC	0.068	0.728	0.274		0.773		0.545	0.480
ER- BC	0.187	0.750	0.152		0.863		0.499	0.515

ER + BC: BC: breast cancer; ER + BC: estrogen positive breast cancer; ER- BC: estrogen negative breast cancer; AM: ang migraine; MA: migraine with aura; MO: migraine without aura. P_(Heterogeneity)_: p value of Cochrane’s Q value in heterogeneity test; P_(Pleiotropy)_: The P value for the intercept in the MR-Egger regression was used present the pleiotropy (*p* < 0.05)

### The effect of MA on breast cancer

In our MR analysis, only three SNPs were selected as IVs at the criteria of *P* < 5 × 10 − 8, and the results showed no causal effect of MA on overall breast cancer (IVW: OR = 0.922, 95% CI = 0.840–1.103, *P* = 0.0919; WM: OR = 0.933, 95% CI = 0.835–1.043, *P* = 0.2253; MR-Egger: OR = 0.926, 95% CI = 0.051–16.97, *P* = 0.9672), ER + breast cancer (IVW: OR = 0.939, 95% CI = 0.840–1.051, *P* = 0.2739; WM: OR = 0.921, 95% CI = 0.809–1.084, *P* = 0.2127; MR-Egger: OR = 0.817, 95% CI = 0.056–58.71, *P* = 0.7931) or ER- breast cancer (IVW: OR = 0.883, 95% CI = 0.745–1.047, *P* = 0.1518; WM: OR = 0.911, 95% CI = 0.740–1.121, *P* = 0.3763; MR-Egger: OR = 0.629, 95% CI = 0.003–119.2, *P* = 0.8907). The MR estimates of the effect of AM on breast cancer for different methods are presented in Supplementary Table 5. The MR-RAPS method was not available because this method was applicable when the sample size of SNPs was greater than 7, but there were only 3 SNPs as IVs for MA exposure. In this part of the sensitivity analysis, we did not find any horizontal pleiotropy or significant heterogeneity (Table 3). We did not detect any MRPRESSO outliers. The plots of the leave-one-out test presented no potentially influential SNPs in this part (Supplementary Fig. 3). Therefore, we can draw a robust conclusion.

### The effect of MO on breast cancer

In the two-sample MR analysis, we found that MO (per log-odds ratio increase) was associated with a 4.2% higher risk of overall breast cancer (IVW: OR = 1.042, 95% CI = 1.005–1.081, *P* = 0.0267; WM: OR = 1.030, 95% CI = 0.977–1.086, *P* = 0.2741; MR-Egger: OR = 1.012, 95% CI = 0.777–1.235, *P* = 0.5324; MR-RAPS: OR = 1.041, 95% CI = 1.003–1.082, *P* = 0.0332) and an 8.9% higher risk of ER- breast cancer (IVW: OR = 1.089, 95% CI = 1.019–1.163, *P* = 0.0118; WM: OR = 1.037, 95% CI = 0.948–1.133, *P* = 0.4313; MR-Egger: OR = 1.102, 95% CI = 0.700-1.354, *P* = 0.8782; MR-RAPS: OR = 1.085, 95% CI = 1.013–1.163, *P* = 0.0205). No causal effect of AM on ER + breast cancer was found (IVW: OR = 1.022, 95% CI = 0.979–1.068, *P* = 0.3217; WM: OR = 1.003, 95% CI = 0.940–1.053, *P* = 0.8582; MR-Egger: OR = 1.002, 95% CI = 0.760–1.272, *P* = 0.6136; MR-RAPS: OR = 1.026, 95% CI = 0.982–1.722, *P* = 0.2556). MO might elevate the risk of ER- breast cancer after FDR control (*FDR* = 0.0354). The effect estimations from various sensitive analysis all pointed to the same direction, which indicated that the results are robust. However, MO was suggestively associated with the risk of overall breast cancer (*FDR* > 0.05 and IVW *P* < 0.05). Detailed information on the MR analysis for different steps is shown in Supplementary Table 6.

No horizontal pleiotropy or heterogeneity was detected in this part of the MR analysis (Table 3). We did not detect any MRPRESSO outliers. Moreover, the leave-one-out test showed that there were potentially influential SNPs in this part (Supplementary Fig. 4). Thus, we should carefully interpret the conclusion.

## Discussion

Our study is the first to investigate the relationship between migraine and breast cancer risk using MR analysis. In our research, we found that migraine prevalence is positively associated with breast cancer. We especially drew the robust conclusion that AM may increase the risk of overall breast cancer or ER- breast cancer, and MO was the risk factor for ER- breast cancer. Additionally, we deemed that MO was suggestively associated with the risk of overall breast cancer because the corrected *FDR* was more than 0.05.

Although all of the above MR‒Eggers suggest no statistical significance, MR‒Egger has the characteristics of inaccuracy and low statistical power, and it is provided as the primary reference result only when horizontal pleiotropy exist. In this study, IVW was applied as the main result, while other sensitivity analysis methods only required consistency of the direction rather than the significance of estimates [28].

Migraine is a common primary headache that occurs more frequently in women than in men [3, 29, 30]. The concentration of estrogen is the main trigger for migraine headaches, and breast cancer is also associated with changes in estrogen [31]. Therefore, there may be a link between migraine and breast cancer [9]. The first clinically controlled study of migraine and breast cancer was published in 2008. Mathes et al. suggested that women who have had migraines in the past have a lower risk of invasive breast cancer in the future (OR, 0.67; 95% CI, 0.54–0.82) [32]. However, Mathes et al. also believed that they did not collect data on the use of NSAIDs, which has been shown in many studies to reduce the incidence of breast cancer [33–35]. There is also an explanation that patients with a history of migraine avoid migraine triggers (i.e., cigarette smoking, alcohol, stress, poor sleep), and some may increase the breast cancer risk [30, 36–39]. In addition, migraine was not associated with breast cancer risk, as reported by a 2015 meta-analysis of 115,378 Nurses Health Studies (HR = 0.95, 95% CI = 0.87 to 1.04) [11]. A recent observational study with a follow-up of up to 7.3 years concluded that women who see more than 4 medical visits per year for migraines tend to be at a nearly twofold higher risk of breast cancer than the control cohort [12]. Although most observational studies suggest that migraine sufferers have a lower risk of breast cancer, migraine and breast cancer, the mechanism of action of estrogen is very complex, which makes the relationship between them unclear [40]. Possible reasons for conflicting conclusions include those studies being conducted in different regions, and they may have different confounding factors affecting breast cancer risk; for example, in women with migraine, independent risk factors for breast cancer include age and alcohol-related disorders, and independent protective factors include the use of antihypertensive drugs, statins, and NSAIDs [12]. In addition, Fan et al. suggested that breast cancer patients may have underestimated the history of migraine because of recall bias, and unmeasured selection bias and confounding factors may have influenced the results [12]. Observational studies are challenging to avoid, and MR studies use genetic proxy tools to study the relationship between exposure and outcome, which can largely avoid this effect. In addition, this study excluded some IVs related to confounding factors by performing the PhenoScanner GWAS datasets. Another strength of our MR study was that two large sample size European datasets were used for SNP-longevity associations. Moreover, there were no indications of pleiotropy or heterogeneity observed in the sensitivity analysis at the last step. We deem that our results differ from those of most previous observational studies because our MR studies can avoid selection bias and confounding factors, which may be unmeasured for observational studies.

It is worth noting that the findings of this study are difficult to interpret as the subtype analysis shows conflicting results when the sample of another subtype is added. For example, AM was associated with a higher risk of ER + breast cancer, but not ER- breast cancer. Some observational studies also shown that the relationship between migraine and breast cancer differed according to hormone receptor status [10, 32]. A meta-analysis about the relationship between migraine and breast cancer also found that there is some subtle heterogeneity by hormone-receptor status of breast cancer [11]. The findings of these observational studies are consistent with our MR analysis.

The mechanisms by which migraine might increase breast cancer risk are probably multifactorial. Migraine patients are more likely to have negative moods, anxiety, insomnia and even depression [41, 42], which are risk factors for breast cancer [43–45]. In addition, Migraine patients tend to reduce physical activity and increase sedentary time to reduce symptoms during a headache attack [46]. But these life behaviors can increase breast cancer risk [47–49].

There are still several limitations in our study. First, most migraine diagnoses are self-reported. Therefore, we cannot rule out misdiagnosis; for example, tension headaches are reported as migraine. Second, we were unable to obtain complete GWAS data of migraine due to the data limitations, so we were unable to perform multivariate MR to adjusted potential confounders, and we were still unable to perform the LDSC to make the genetic correlations of migraine and breast cancer, but we excluded confounder-related IVs as much as possible by browsing the PhenoScanner GWAS datasets. We can believe that the independent genetic variants related to migraine were reliable due to the rigorous investigation on the GWAS study. The lack of multivariate MR analysis should not diminish the role of migraine as etiological for breast cancer. Women with a history of migraine should strengthen breast cancer screening to be diagnosed early and treated adequately. Third, our outcomes and exposures are all of European descent and lack universality. Research on other races will be necessary for the future because of the high heterogeneity of migraine and breast cancer. Forth, we cannot rule out the existence of potential horizontal pleiotropy leading to biased results in MR studies, although comprehensive array of sensitivity analysis didn’t detect any pleiotropy. Fifth, we could not evaluate the nonlinear associations between migraine and breast cancer without individual-level data. In addition, the relatively small phenotypic variance of MA (approximately 0.9%) due to only 3 SNPs were selected, which requires larger GWAS studies to obtain more genetic data in the future study.

## Conclusion

Previous observational studies have been controversial as to whether women with a history of migraine have an increased risk of breast cancer. We, as the first study to utilize MR analysis, also made significant discoveries. Our study found that migraine may be a risk factor for overall breast cancer and ER + breast cancer but not ER- breast cancer. In subtype analysis, MO may increase the risk of ER- breast cancer and is suggestively associated with the risk of overall breast cancer but not ER + breast cancer. No evidence to support the association of MA with breast cancer and its subtypes. Therefore, women with a history of migraine, especially MO, should strengthen breast cancer screening to be diagnosed early and treated adequately.

### Electronic supplementary material

Below is the link to the electronic supplementary material.

Supplementary Material 1

Supplementary Material 2

Supplementary Material 3

Supplementary Material 4

Supplementary Material 5

Supplementary Material 6

Supplementary Material 7

Supplementary Material 8

## Data Availability

The data of breast cancer can be obtained in MRCIEU (https://gwas.mrcieu.ac.uk/; ieu-a-1126; ieu-a-1127; ieu-a-1128). The data of IVs for migraine is reported in the supplementary tables of the published papers (DOI: 10.1038/s41588-021-00990-0). The code used in this article was shown in the Supplementary File Code.

## Abbreviations

MR: Mendelian randomization
GWAS: Genome-wide association studies
IVW: Inverse variant weighted
MR-PRESSO: MR pleiotropy residual sum and outlier
MR-RAPS: MR-Robust Adjusted Profile Score
AM: Any migraine
MO: Migraine without aura
MA: Migraine with aura
OR: Odds ratio
CI: Confidence intervals
FDR: False discovery rate
ER+: Estrogen receptor-positive
ER-: Estrogen receptor-negative
NSAIDs: Nonsteroidal anti-inflammatory drugs
SNPs: Single nucleotide polymorphisms
IVs: Instrument variants
LD: Linkage disequilibrium
BMI: Body mass index
WM: Weighted median
