# Supplementary material for: Effect of genetic liability to migraine and its subtypes on breast cancer: a mendelian randomization study
Source: BMC Cancer. 2023 Sep 20;23:887. doi: 10.1186/s12885-023-11337-9 (PMC10510189; doi:10.1186/s12885-023-11337-9)
Supplement: Supplementary file 1 — Supplementary Material 1 [file 12885_2023_11337_MOESM1_ESM.pdf]

Supplementary Table 1: Summary of the three methods used for MR analysis

Method	Assumptions	Strengths
The inverse variance weighted method	No unbalanced horizontal pleiotropy	Provides the most precise estimate if all instruments are valid
The weighted median method	More than 50% of weight from valid genetic instruments	Informs about the estimate supported by the majority of evidence
MR-Egger regression	Associations of the genetic instruments with the exposure are uncorrelated with any pleiotropic effects of the instruments on the outcome	Provides consistent estimate under this assumption
MR-RAPS	RAPS considers the measurement error in SNP-exposure effects and the biases caused by weak instruments, and is robust for alleviating pleiotropy.	Overcome the bias of weak instrumental variables and the effects of systematic and idiosyncratic pleiotropy to obtain a robust causal assessment.
MR-PRESSO	The largest group of candidate instruments with similar estimates is the group of valid instrumental variables.	Detects outliers and provides estimate after removal of outliers
