# Supplementary material for: Effect of genetic liability to migraine and its subtypes on breast cancer: a mendelian randomization study
Source: BMC Cancer. 2023 Sep 20;23:887. doi: 10.1186/s12885-023-11337-9 (PMC10510189; doi:10.1186/s12885-023-11337-9)
Supplement: Supplementary file 2 — Supplementary Material 2 [file 12885_2023_11337_MOESM2_ESM.pdf]

Supplementary Table 4: MR estimates for the causal effect of AM on BC step by step.

Outcome	Step#	NSNP	IVW		$P(I^2)$	MRPRESSO	Weighted median		MR-Egger		$P_{(intercept)}$	MR-RAPS	
			OR (95%CI)	P		$P_{(Global)}$	OR (95%CI)	P	OR (95%CI)	P		OR (95%CI)	P
Overall BC	1	84	1.064 (1.018, 1.113)	**0.0058**	0.0001	0.0003	1.062 (1.005, 1.121)	**0.0327**	1.047 (0.927, 1.183)	0.4698	0.7822	1.065 (1.018, 1.115)	**0.0060**
	2	82	1.077 (1.034, 1.122)	**0.0004**	0.0064	0.0057	1.064 (1.007, 1.123)	**0.0274**	1.055 (0.942, 1.181)	0.3568	0.6948	1.072 (1.027, 1.120)	**0.0017**
	3	79	1.072 (1.035, 1.110)	**8.78×10^{-5}**	0.0835	0.1040	1.063 (1.006, 1.123)	**0.0283**	1.062 (0.956, 1.179)	0.2639	0.8458	1.069 (1.026, 1.113)	**0.0015**
ER+ BC	1	84	1.062 (1.010, 1.116)	**0.0177**	0.0026	NA	1.006 (0.944, 1.073)	0.8483	1.032 (0.900, 1.183)	0.6535	0.6606	1.059 (1.006, 1.115)	**0.0276**
	3	81	1.066 (1.023, 1.111)	**0.0024**	0.0680	0.0710	1.010 (0.947, 1.078)	0.7533	1.038 (0.902, 1.160)	0.7277	0.4897	1.064 (1.013, 1.117)	**0.0128**
ER- BC	1	84	1.062 (0.987, 1.143)	0.1078	0.0093	NA	1.037 (0.940, 1.144)	0.4728	1.077 (0.880, 1.319)	0.4714	0.8801	1.059 (0.982, 1.142)	0.1357
	3	80	1.045 (0.979, 1.115)	0.1871	0.3348	0.3382	1.036 (0.939, 1.144)	0.4784	1.073 (0.899, 1.281)	0.4368	0.7499	1.050 (0.980, 1.126)	0.1658

Step#: 1, MR analysis with the all remained SNPs; 2, MR analysis after eliminating MRPRESSO outlier (with $P < 0.05$); 3, MR analysis after removing all the SNPs (with $P < 1.00$ in MR-PRESSO test); NSNP, number of single nucleotide polymorphism; MR, Mendelian randomization; IVW: inverse variance weighting; MR-RAPS: Robust Adjusted Profile Score; BC: breast cancer; ER+ BC: estrogen receptor positive breast cancer; ER- BC: estrogen receptor negative breast cancer; AM: any migraine; OR: odds ratio; The I^2 statistic was used to present the heterogeneity among estimates for each SNPs in one analysis; $P_{(Global)}$: The p value for the global test in the MR-PRESSO; $P_{(intercept)}$: The p value for the intercept in the MR-Egger regression was used present the pleiotropy (p<0.05); Bold font: The p-values < 0.05 are statistically significant. The ORs were scaled to a 1-unit increase in log-transformed OR of migraine. P values are for ORs (95% CIs).
