# Supplementary material for: Effect of genetic liability to migraine and its subtypes on breast cancer: a mendelian randomization study
Source: BMC Cancer. 2023 Sep 20;23:887. doi: 10.1186/s12885-023-11337-9 (PMC10510189; doi:10.1186/s12885-023-11337-9)
Supplement: Supplementary file 3 — Supplementary Material 3 [file 12885_2023_11337_MOESM3_ESM.pdf]

Supplementary Table 3: Power estimation of this Mendelian randomization analysis*

Disease	Sample size	Proportion of cases	OR with 80% power in analysis of AM	OR with 80% power in MA	OR with 80% power in MO
Breast cancer	228,951	0.537	1.046	1.132	1.040
ER+ Breast cancer	175,475	0.396	1.052	1.155	1.050
ER- Breast cancer	127,442	0.168	1.082	1.248	1.075

* Type 1 error of 5% and explained variance for AM, MA, MO were 6.93%, 0.91%, and 8.26% respectively.
ER+: estrogen receptor positive; ER-: estrogen receptor negative; AM: any migraine; OR: odds ratio; MA: migraine with aura; MO: migraine without aura.
