# Supplementary material for: Effect of genetic liability to migraine and its subtypes on breast cancer: a mendelian randomization study
Source: BMC Cancer. 2023 Sep 20;23:887. doi: 10.1186/s12885-023-11337-9 (PMC10510189; doi:10.1186/s12885-023-11337-9)
Supplement: Supplementary file 4 — Supplementary Material 4 [file 12885_2023_11337_MOESM4_ESM.pdf]

Supplementary Table 2: Detail information of selected IVs.

Exposure	SNP	CHR	Position	EA	NEA	beta	se	eaf	p	F
AM	rs1003194	11	15126085	A	G	0.0340	0.0054	0.383	2.43E-10	39.6
AM	rs10038882	5	145752008	T	C	0.0420	0.0060	0.748	1.33E-12	49.0
AM	rs10166942	2	234825093	T	C	0.0990	0.0066	0.805	9.35E-51	225.0
AM	rs10218452	1	3075597	G	A	0.1100	0.0062	0.229	7.26E-71	314.8
AM	rs10234636	7	40427617	T	C	0.0890	0.0081	0.111	4.43E-28	120.7
AM	rs10405121	19	13339128	G	A	0.0330	0.0053	0.548	4.74E-10	38.8
AM	rs10456100	6	39183470	T	C	0.0510	0.0057	0.281	9.16E-19	80.1
AM	rs10894756	11	133745852	G	A	0.0290	0.0053	0.573	2.83E-08	29.9
AM	rs11031122	11	30547438	C	T	0.0370	0.0059	0.243	6.91E-10	39.3
AM	rs11153082	6	97059666	G	A	0.0840	0.0054	0.331	7.26E-54	242.0
AM	rs11165300	1	92177663	G	T	0.0330	0.0060	0.239	4.72E-08	30.3
AM	rs11487328	1	174601659	G	C	0.0480	0.0086	0.746	1.7E-08	31.2
AM	rs11624776	14	93595591	A	C	0.0500	0.0056	0.684	9.75E-19	79.7
AM	rs11782789	8	27266287	A	T	0.0410	0.0070	0.836	3.03E-09	34.3
AM	rs12057629	1	15538493	C	T	0.0400	0.0054	0.356	9.38E-14	54.9
AM	rs12226331	11	102070976	T	A	0.0400	0.0054	0.348	1.92E-13	54.9
AM	rs12260159	10	100702737	G	A	0.0830	0.0103	0.925	7.33E-16	64.9
AM	rs12260436	10	104741114	C	A	0.0360	0.0058	0.257	7.29E-10	38.5
AM	rs12452590	17	60720058	G	T	0.0380	0.0059	0.375	2.03E-10	41.5
AM	rs1245463	14	27661650	A	G	0.0400	0.0053	0.388	5.72E-14	57.0
AM	rs12598836	16	4534482	G	A	0.0380	0.0059	0.304	2.21E-10	41.5
AM	rs12712881	2	43649780	A	C	0.0330	0.0052	0.435	3.5E-10	40.3
AM	rs13078967	3	154289946	A	C	0.1460	0.0178	0.973	2.16E-16	67.3
AM	rs13235543	7	73013901	C	T	0.0580	0.0080	0.873	3.06E-13	52.6
AM	rs138556413	2	203832867	C	T	0.1290	0.0159	0.964	4.15E-16	65.8
AM	rs1499963	3	124607055	C	T	0.0320	0.0056	0.679	7.48E-09	32.7
AM	rs1542668	14	42548912	G	A	0.0310	0.0055	0.671	2.53E-08	31.8
AM	rs17723637	9	109687403	G	A	0.0410	0.0072	0.150	8.63E-09	32.4
AM	rs1982072	19	41864509	A	T	0.0370	0.0056	0.700	4.22E-11	43.7
AM	rs2000660	13	110788441	A	G	0.0500	0.0091	0.092	4.95E-08	30.2
AM	rs2078371	1	115677183	C	T	0.1060	0.0078	0.117	5.87E-42	184.7
AM	rs2119930	17	47514039	G	T	0.0410	0.0052	0.405	6.69E-15	62.2
AM	rs2274319	1	156450873	T	C	0.0730	0.0054	0.347	2.74E-41	182.8
AM	rs246326	5	122306398	T	C	0.0480	0.0077	0.126	6.8E-10	38.9
AM	rs2672592	10	124230750	T	G	0.0380	0.0054	0.363	1.22E-12	49.5
AM	rs28451064	21	35593827	G	A	0.0630	0.0080	0.869	3.52E-15	62.0
AM	rs28455731	6	121846038	T	G	0.0690	0.0070	0.157	8.82E-23	97.2
AM	rs28739509	1	38366907	C	T	0.0390	0.0061	0.273	2.64E-10	40.9
AM	rs28756401	14	58761912	G	A	0.0330	0.0058	0.715	6.4E-09	32.4
AM	rs34273564	6	72321017	T	C	0.0340	0.0052	0.481	1E-10	42.8
AM	rs3891689	9	119258583	C	T	0.0580	0.0061	0.234	2.28E-21	90.4

AM	rs4278223	9	140743200	T	A	0.0450	0.0073	0.636	6.24E-10	38.0
AM	rs4668251	2	171234235	G	C	0.0330	0.0058	0.710	7.58E-09	32.4
AM	rs4705403	5	149380493	A	G	0.0470	0.0083	0.104	1.18E-08	32.1
AM	rs4739105	8	64496159	T	C	0.0360	0.0064	0.211	2.85E-08	31.6
AM	rs4814864	20	19469817	C	G	0.0650	0.0058	0.254	1.44E-28	125.6
AM	rs4907224	2	96576609	A	T	0.0360	0.0059	0.676	1.63E-09	37.2
AM	rs4910165	11	10674044	G	C	0.0570	0.0055	0.678	1.09E-24	107.4
AM	rs55707505	14	75362552	T	C	0.0310	0.0055	0.675	2.48E-08	31.8
AM	rs56067931	7	120481569	C	T	0.0360	0.0065	0.800	4.83E-08	30.7
AM	rs56140113	1	206843108	C	T	0.0370	0.0064	0.780	7.76E-09	33.4
AM	rs580845	9	14103618	A	C	0.0300	0.0055	0.601	4.3E-08	29.8
AM	rs6057599	20	31168439	T	C	0.0410	0.0055	0.335	8.73E-14	55.6
AM	rs625686	22	20142932	C	T	0.0340	0.0059	0.306	8.26E-09	33.2
AM	rs6556059	5	172645766	T	C	0.0340	0.0055	0.363	8.16E-10	38.2
AM	rs6568677	6	111713302	A	G	0.0350	0.0063	0.209	2.09E-08	30.9
AM	rs6668908	1	186913055	G	T	0.0310	0.0055	0.669	2.22E-08	31.8
AM	rs6795209	3	88210464	A	G	0.0410	0.0072	0.191	1.23E-08	32.4
AM	rs7034179	9	71746838	T	C	0.0430	0.0052	0.429	1.6E-16	68.4
AM	rs72764846	1	245847455	G	A	0.0380	0.0064	0.784	5.41E-09	35.3
AM	rs72923449	2	176978383	C	A	0.0780	0.0142	0.036	4.66E-08	30.2
AM	rs7335684	13	47193696	G	A	0.0340	0.0060	0.247	1.05E-08	32.1
AM	rs7371912	3	30472786	A	G	0.0440	0.0057	0.303	1.06E-14	59.6
AM	rs73805934	4	35469918	G	C	0.0420	0.0069	0.823	1.11E-09	37.1
AM	rs74182632	19	19406126	A	G	0.0640	0.0112	0.055	1.43E-08	32.7
AM	rs7511672	1	66178918	G	A	0.0310	0.0052	0.538	1.43E-09	35.5
AM	rs764508	21	36935896	C	T	0.0320	0.0053	0.370	3.28E-09	36.5
AM	rs7684253	4	57727311	T	C	0.0390	0.0052	0.550	4.21E-14	56.3
AM	rs7916911	10	8722944	T	G	0.0400	0.0057	0.284	3.18E-12	49.2
AM	rs7932866	11	46548094	A	G	0.0430	0.0072	0.836	2.38E-09	35.7
AM	rs7996252	13	78876537	T	C	0.0290	0.0053	0.596	4.11E-08	29.9
AM	rs8046696	16	75442143	T	G	0.0410	0.0055	0.428	4.76E-14	55.6
AM	rs8052831	16	87578039	G	A	0.0430	0.0055	0.344	8.25E-15	61.1
AM	rs8077768	17	78256432	C	T	0.0400	0.0056	0.477	9.32E-13	51.0
AM	rs8087942	18	55192245	A	G	0.0390	0.0055	0.667	9.71E-13	50.3
AM	rs843215	2	156416638	G	A	0.0290	0.0051	0.468	2.61E-08	32.3
AM	rs869432	10	112502662	A	C	0.0290	0.0053	0.584	3.54E-08	29.9
AM	rs895219	2	146037564	C	T	0.0370	0.0056	0.300	3.74E-11	43.7
AM	rs910187	20	45841052	G	A	0.0350	0.0054	0.628	1.14E-10	42.0
AM	rs9295536	6	22131929	C	A	0.0360	0.0052	0.562	7.75E-12	47.9
AM	rs9349379	6	12903957	A	G	0.0770	0.0053	0.590	1.41E-47	211.1
AM	rs9383843	6	150133954	C	A	0.0330	0.0055	0.647	1.35E-09	36.0
AM	rs9468830	6	30749712	T	G	0.0360	0.0064	0.706	2.38E-08	31.6
AM	rs9894634	17	1967501	C	T	0.0340	0.0052	0.403	9.64E-11	42.8
MA	rs11153082	6	97059666	G	A	0.0891	0.0140	0.325	1.99E-10	40.5

MA	rs11031122	11	30547438	C	T	0.0881	0.0151	0.233	6.45E-09	33.7
MA	rs12598836	16	4534482	G	A	0.0827	0.0148	0.294	2.81E-08	30.9
MO	rs11153082	5	145752008	G	A	0.3249	0.1353	0.014	2.38E-21	90.1
MO	rs9349379	2	234825093	A	G	0.5935	0.1321	0.014	6.31E-20	83.6
MO	rs10166942	7	40427617	T	C	0.7982	0.1512	0.017	4.96E-18	75.0
MO	rs2078371	6	97059666	C	T	0.1155	0.1592	0.020	8.33E-15	60.3
MO	rs3891689	1	115677183	C	T	0.2338	0.1226	0.016	1.52E-14	59.2
MO	rs2274319	1	156450873	T	C	0.3554	0.1084	0.014	1.77E-14	58.9
MO	rs4910165	6	121846038	G	C	0.6715	0.0997	0.015	8.1E-12	46.8
MO	rs28455731	9	119258583	T	G	0.1640	0.1115	0.018	8.36E-10	37.7
MO	rs7684253	20	19469817	T	C	0.5514	0.0815	0.014	2.38E-09	35.7
MO	rs4814864	11	10674044	C	G	0.2494	0.0903	0.015	3.69E-09	34.8
MO	rs10038882	4	57727311	T	C	0.7501	0.0871	0.016	4.13E-08	30.1
MO	rs10234636	6	12903957	T	C	0.1094	0.1165	0.021	4.29E-08	30.1

CHR: Chromosome; EA, effect allele; EAF, effect allele frequency; NEA, non-effect allele; se, standard error; AM: any migraine; MA: migraine with aura; MO: migraine without aura.
