# Supplementary material for: Effect of genetic liability to migraine and its subtypes on breast cancer: a mendelian randomization study
Source: BMC Cancer. 2023 Sep 20;23:887. doi: 10.1186/s12885-023-11337-9 (PMC10510189; doi:10.1186/s12885-023-11337-9)
Supplement: Supplementary file 5 — Supplementary Material 5 [file 12885_2023_11337_MOESM5_ESM.pdf]

Supplementary Table 5: MR estimates for the causal effect of MA on BC.

Outcome	NSNP	IVW		*P*(I^2)	Weighted median		MR-Egger		*P*($_{intercept}$)	MR-RAPS&
		OR (95%CI)	*P*		OR (95%CI)	*P*	OR (95%CI)	*P*		
Overall BC	3	0.922 (0.840, 1.103)	0.0919	0.7653	0.933 (0.835, 1.043)	0.2253	0.926 (0.051, 16.97)	0.9672	0.9982	-
ER+ BC	3	0.939 (0.840, 1.051)	0.2739	0.7992	0.921 (0.809, 1.048)	0.2127	0.817 (0.056, 58.71)	0.7931	0.7730	-
ER- BC	3	0.883 (0.745, 1.047)	0.1518	0.9197	0.911 (0.740, 1.121)	0.3763	0.629 (0.003, 119.2)	0.8907	0.8627	-

NSNP, number of single nucleotide polymorphism; MR, Mendelian randomization; IVW: inverse variance weighting; BC: breast cancer; ER+ BC: estrogen receptor positive breast cancer; ER- BC: estrogen receptor negative breast cancer; MA: migraine with aura; OR: odds ratio; The I^2 statistic was used to present the heterogeneity among estimates for each SNPs in one analysis; *P*($_{intercept}$): The *p* value for the intercept in the MR-Egger regression was used present the pleiotropy (p<0.05). MR-RAPS&: Robust Adjusted Profile Score (number of SNP must be greater than 7); Bold font: The p-values < 0.05 are statistically significant. The ORs were scaled to a 1-unit increase in log-transformed OR of migraine. P values are for ORs (95% CIs).
