# Supplementary material for: Effect of genetic liability to migraine and its subtypes on breast cancer: a mendelian randomization study
Source: BMC Cancer. 2023 Sep 20;23:887. doi: 10.1186/s12885-023-11337-9 (PMC10510189; doi:10.1186/s12885-023-11337-9)
Supplement: Supplementary file 6 — Supplementary Material 6 [file 12885_2023_11337_MOESM6_ESM.pdf]

Supplementary Table 6: MR estimates for the causal effect of MO on BC step by step.

Outcome	Step[#]	NSNP	IVW		$P(I^2)$	MRPRESSO	Weighted median		MR-Egger		$P_{(intercept)}$	MR-RAPS	
			OR (95%CI)	*P*		$P_{(Global)}$	OR (95%CI)	*P*	OR (95%CI)	*P*		OR (95%CI)	*P*
Overall BC	1	12	1.042 (1.005, 1.081)	**0.0267**	0.3473	0.427	1.030 (0.977, 1.086)	0.2741	1.012 (0.777, 1.235)	0.5324	0.2998	1.041 (1.003, 1.082)	**0.0332**
ER+ BC	1	12	1.022 (0.979, 1.068)	0.3217	0.5453	0.529	1.003 (0.940, 1.053)	0.8582	1.002 (0.760, 1.272)	0.6136	0.4795	1.026 (0.982, 1.722)	0.2556
ER+ BC	1	12	1.089 (1.019, 1.163)	**0.0118**	0.4985	0.479	1.037 (0.948, 1.133)	0.4313	1.102 (0.700, 1.354)	0.8782	0.5149	1.085 (1.013, 1.163)	**0.0205**

Step[#]: 1, MR analysis with the all remained SNPs; 2, MR analysis after eliminating MRPRESSO outlier (with $P < 0.05$); 3, MR analysis after removing all the SNPs (with $P < 1.00$ in MR-PRESSO test); Se, standard error; SNP, single nucleotide polymorphism; MR, Mendelian randomization; IVW: inverse variance weighting; MR-RAPS: Robust Adjusted Profile Score; BC: breast cancer; ER+ BC: estrogen receptor positive breast cancer; ER- BC: estrogen receptor negative breast cancer; MO: migraine without aura; OR: odds ratio; The I^2 statistic was used to present the heterogeneity among estimates for each SNPs in one analysis; $P_{(Global)}$: The *p* value for the global test in the MR-PRESSO; $P_{(intercept)}$: The *p* value for the intercept in the MR-Egger regression was used present the pleiotropy ($p<0.05$); Bold font: The p-values < 0.05 are statistically significant. The ORs were scaled to a 1-unit increase in log-transformed OR of migraine. P values are for ORs (95% CIs).
