# Supplementary material for: Effect of genetic liability to migraine and its subtypes on breast cancer: a mendelian randomization study
Source: BMC Cancer. 2023 Sep 20;23:887. doi: 10.1186/s12885-023-11337-9 (PMC10510189; doi:10.1186/s12885-023-11337-9)
Supplement: Supplementary file 7 — Supplementary Material 7 [file 12885_2023_11337_MOESM7_ESM.pdf]

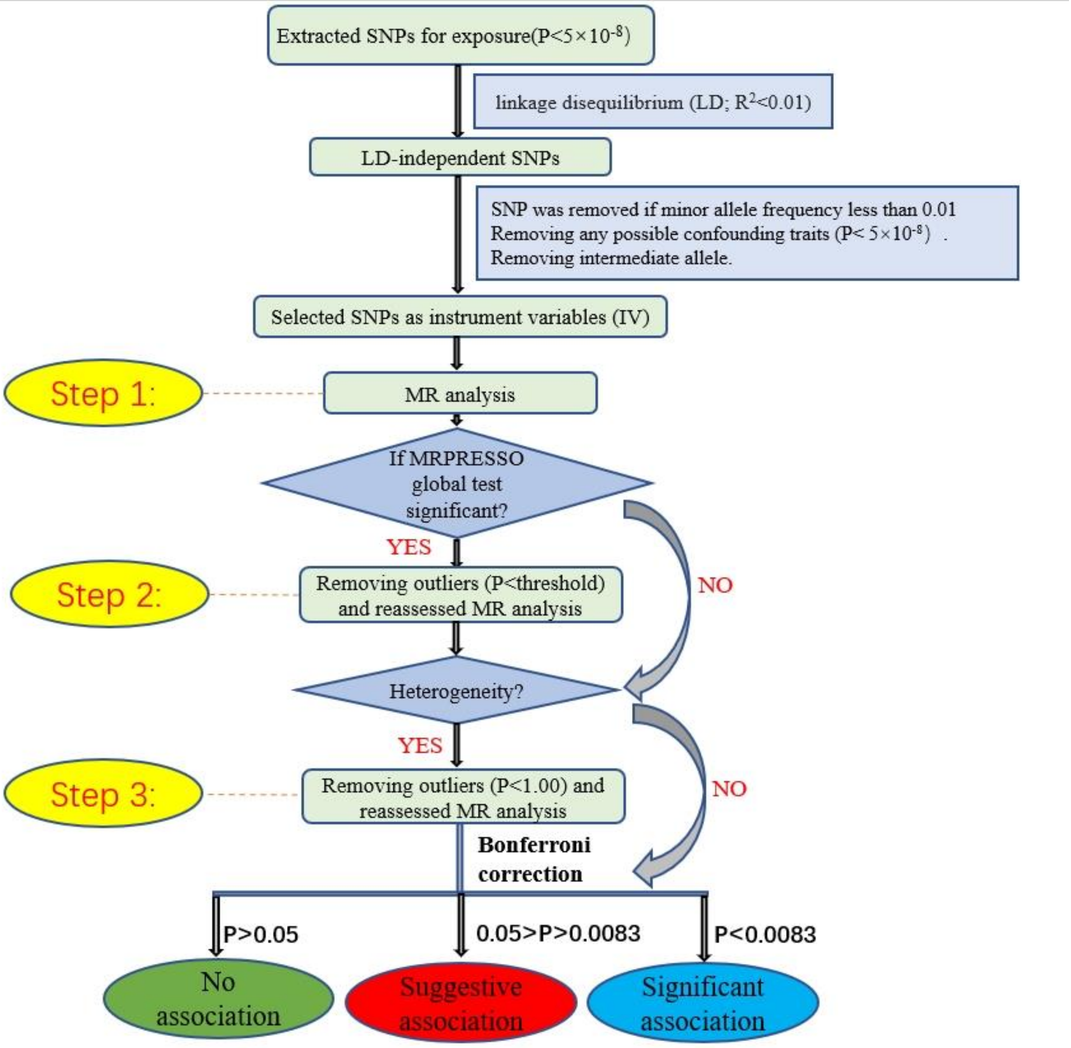

Supplementary Figure 1: Flow chart about the analytical methods and how the MR analysis was performed step-by-step.

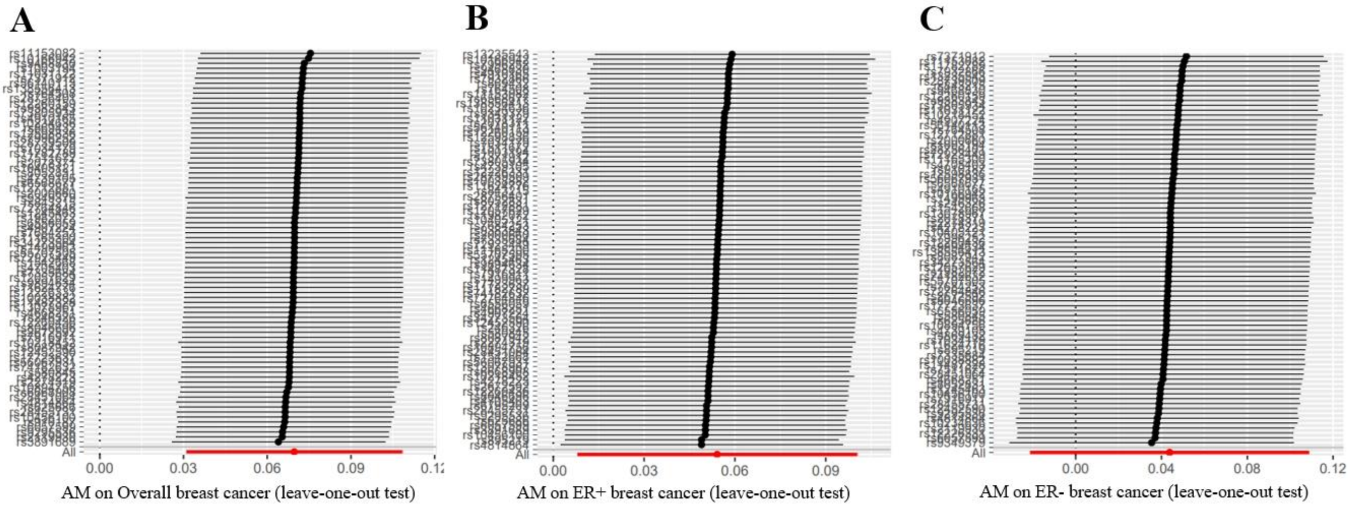

Supplementary Figure 2. Leave-one-out plots in the Mendelian randomization analysis of AM on overall breast cancer (A), ER+ breast cancer (B) and ER- breast cancer risk.

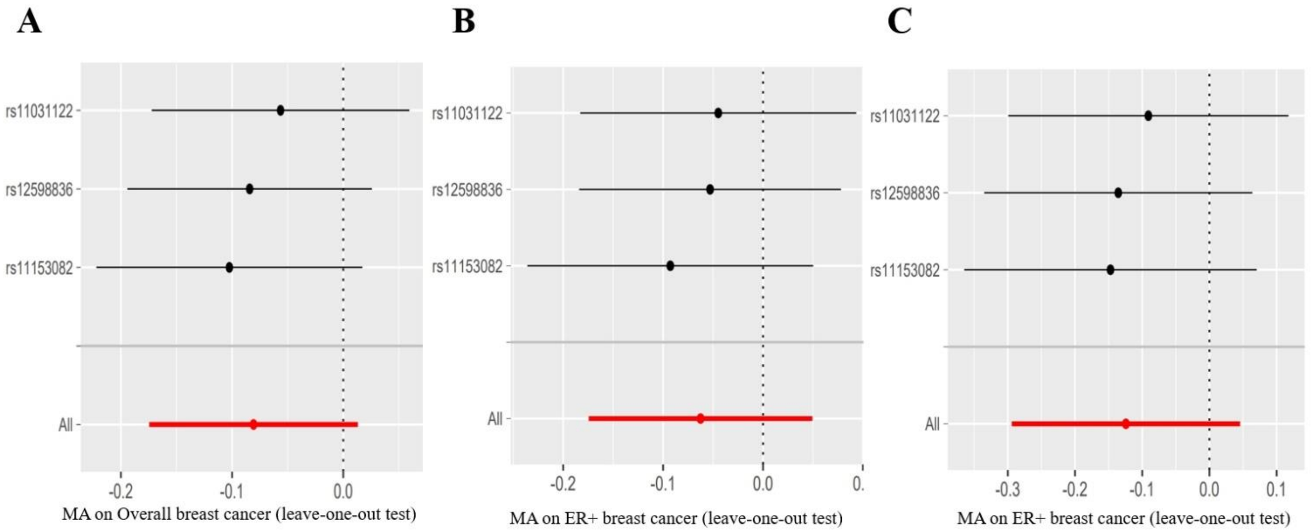

Supplementary Figure 3. Leave-one-out plots in the Mendelian randomization analysis of MA on overall breast cancer (A), ER+ breast cancer (B) and ER- breast cancer risk.

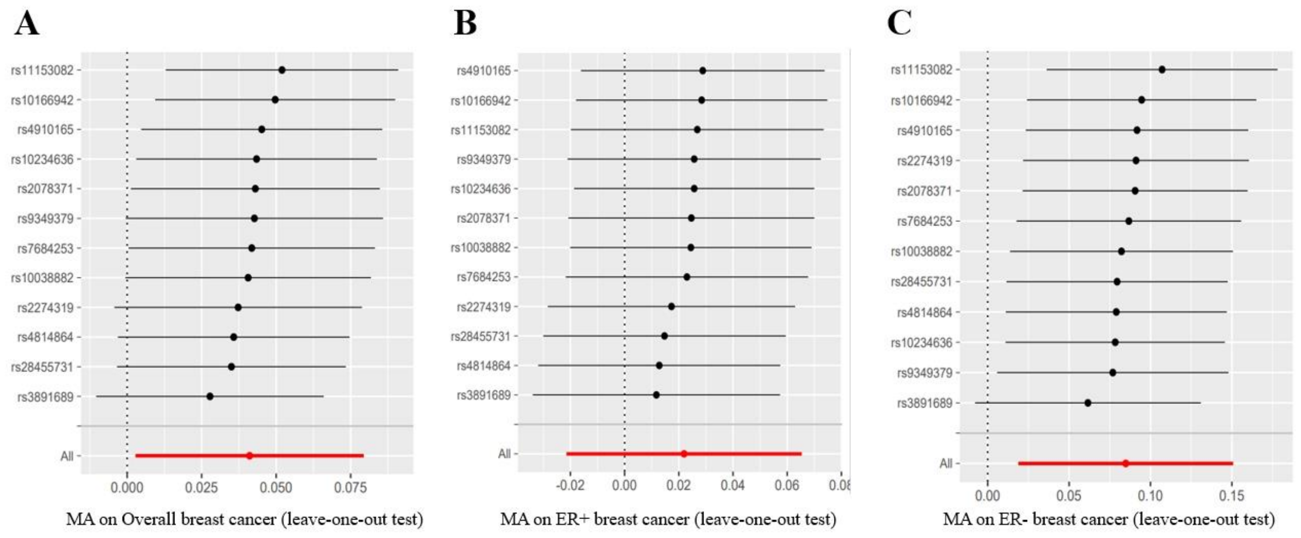

Supplementary Figure 4. Leave-one-out plots in the Mendelian randomization analysis of MO on overall breast cancer (A), ER+ breast cancer (B) and ER- breast cancer risk.
