# Supplementary material for: Effect of genetic liability to migraine and its subtypes on breast cancer: a mendelian randomization study
Source: BMC Cancer. 2023 Sep 20;23:887. doi: 10.1186/s12885-023-11337-9 (PMC10510189; doi:10.1186/s12885-023-11337-9)
Supplement: Supplementary file 8 — Supplementary Material 8 [file 12885_2023_11337_MOESM8_ESM.pdf]

Supplementary File Code: The code used in this article.

```
library(TwoSampleMR)
aa <-read.table('expousure.txt',header=T)
exp_dat <- format_data(
aa,
  type='exposure',
  phenotype_col = "phenotype",
  snp_col = "SNP",
  beta_col = "beta",
  se_col = "se",
  effect_allele_col ="a1",
  other_allele_col = "a2",
  eaf_col = "eaf",
  pval_col = "p" )

out_dat <-read.table('outcome.txt',header=T)
out_dat <- format_data(
  dat=out_dat,
  type = "outcome",
  snps = exp_dat$SNP,
  header = TRUE,
  phenotype_col = "phenotype",
  snp_col = "SNP",
  beta_col = "beta",
  se_col = "se",
  effect_allele_col = "a1",
  other_allele_col = "a2",
  eaf_col = "eaf",
  pval_col = "p",
  ncase_col = "N",
  chr_col = "CHR",
  pos_col = "POS")

mydata <- harmonise_data(
            exposure_dat=exp_dat,
            outcome_dat=out_dat,
            action= 2
            )
res <- mr(mydata)
res

het <- mr_heterogeneity(mydata)
het
```

```
pleio <- mr_pleiotropy_test(mydata)
pleio
library(MRPRESSO)
mr_presso(BetaOutcome ="beta.outcome", BetaExposure = "beta.exposure", SdOutcome ="se.outcome", SdExposure = "se.exposure",
          OUTLIERtest = TRUE,DISTORTIONtest = TRUE, data = mydata, NbDistribution = 1000,
          SignifThreshold = 0.05)

library(mr.raps)
res <- mr.raps(mydata$beta.exposure, mydata$beta.outcome, mydata$se.exposure,
               mydata$se.outcome,   TRUE,
               "tukey", diagnosis = TRUE)
```
